# Modifications of Epitaxial Graphene on SiC for the Electrochemical Detection and Identification of Heavy Metal Salts in Seawater

**DOI:** 10.3390/s22145367

**Published:** 2022-07-19

**Authors:** Jenifer R. Hajzus, Lisa C. Shriver-Lake, Scott N. Dean, Jeffrey S. Erickson, Daniel Zabetakis, Joel Golden, Daniel J. Pennachio, Rachael L. Myers-Ward, Scott A. Trammell

**Affiliations:** 1 American Society for Engineering Education, U.S. Naval Research Laboratory, Washington, DC 20375, USA; jenifer.hajzus.ctr@nrl.navy.mil; 2 U.S. Naval Research Laboratory, 4555 Overlook Avenue SW, Washington, DC 20375, USA; lisa.shriverlake@nrl.navy.mil (L.C.S.-L.); scott.dean@nrl.navy.mil (S.N.D.); jeffrey.erickson@nrl.navy.mil (J.S.E.); daniel.zabetakis@nrl.navy.mil (D.Z.); joel.golden@nrl.navy.mil (J.G.); rachael.myers-ward@nrl.navy.mil (R.L.M.-W.); 3 National Research Council, U.S. Naval Research Laboratory, Washington, DC 20375, USA; daniel.pennachio.ctr@nrl.navy.mil

**Keywords:** cyclic square wave voltammetry, heavy metal ions, identification algorithms, machine learning, modified epitaxial graphene, seawater

## Abstract

The electrochemical detection of heavy metal ions is reported using an inexpensive portable in-house built potentiostat and epitaxial graphene. Monolayer, hydrogen-intercalated quasi-freestanding bilayer, and multilayer epitaxial graphene were each tested as working electrodes before and after modification with an oxygen plasma etch to introduce oxygen chemical groups to the surface. The graphene samples were characterized using X-ray photoelectron spectroscopy, atomic force microscopy, Raman spectroscopy, and van der Pauw Hall measurements. Dose–response curves in seawater were evaluated with added trace levels of four heavy metal salts (CdCl_2_, CuSO_4_, HgCl_2_, and PbCl_2_), along with detection algorithms based on machine learning and library development for each form of graphene and its oxygen plasma modification. Oxygen plasma-modified, hydrogen-intercalated quasi-freestanding bilayer epitaxial graphene was found to perform best for correctly identifying heavy metals in seawater.

## 1. Introduction

The development of miniaturized sensors for electroanalytical measurements continues to be a dynamic field for a variety of analytical applications in marine environments [1,2,3,4,5]. One aim is to integrate inexpensive and low power electrochemical sensors into maritime platforms for the trace analysis of chemicals in seawater to monitor environmental pollutants and hazardous materials that threaten divers engaged in underwater activity [6,7]. Heavy metals, for example cadmium (Cd), mercury (Hg), lead (Pb), copper (Cu), arsenic (As), and chromium (Cr), are a type of potential chemical hazard in aquatic environments that can cause damage to multiple organs and have increased in environmental abundance due to human and industrial activity [8,9]. Certain heavy metals such as Cu are required by the human body in trace amounts but can be toxic in high concentrations, whereas other heavy metals, including Cd, Hg, Pb, As, and Cr, are toxic in small quantities [8,9,10]. Detection of heavy metals to determine potential exposure is therefore crucial. Hg-based working electrodes have traditionally been studied for electrochemical detection of heavy metals, however, global phase-out of Hg-containing products due to environmental and health concerns has motivated the pursuit of alternative electrode materials [11].

Graphene has several unique physical and electrical properties, such as high electron mobility, high mechanical strength, and a high surface-to-volume ratio, which enable its use as a working electrode for various electroanalytical applications [12,13,14,15], including the detection of heavy metal ions in water [14,15,16,17]. While graphene can be synthesized by a variety methods [14], epitaxial graphene (EG) formed by sublimation of silicon (Si) from the Si-face of silicon carbide (SiC(0001)) is particularly attractive because it can be grown as a continuous layer with low defect density over wafer-scale areas, does not require transfer to another substrate, is not prone to aggregation issues [14,18], can be produced with a reproducible surface [11], and is compatible with semiconductor processing methods. By changing growth parameters such as the temperature, pressure, growth duration, and the offcut of the SiC substrate, properties of EG such as the number of graphene layers and surface morphology can be controllably varied. Additionally, EG can be further modified by a hydrogen intercalation process to decouple the 6√3 buffer layer at the SiC(0001) interface, resulting in increased carrier mobilities and the formation of quasi-freestanding (QFS) EG [19].

Recent detailed electrochemical studies of Cd, Hg, Pb, and Cu at nominally monolayer EG/SiC(0001) electrodes combined with density functional theory (DFT) modeling and Raman spectroscopy have provided insight into the adsorption behavior of these heavy metals at EG [20,21,22,23,24,25]. It was found that binding to graphene is stronger for Pb than for Cd and Hg, which interact with graphene through van der Waals forces, suggesting a need to increase the number of electroactive sites to improve graphene sensitivity towards Hg and Cd [20,23,24]. DFT modeling by Elgengehi et al. showed oxygen functionalization of graphene with epoxy and/or hydroxyl groups can enhance the adsorption of Cd and Pb at graphene [26], therefore one approach to improve electroanalytical performance of EG for trace detection may be by surface modification [14] such as a plasma etch to add chemical functional groups at the surface of graphene [27,28,29,30]. The influence of the supporting substrate of graphene may also impact metal adsorption. For example, scanning tunneling microscopy and spectroscopy experiments of cobalt and nickel adatoms on EG on SiC(0001) by Eelbo et al. found the stable adatom configurations were influenced by the degree of decoupling of graphene from its substrate, in which a single stable adsorption site was observed at pristine monolayer EG, whereas two sites were observed at hydrogen-intercalated QFS monolayer EG [31].

We have previously developed a configurable sensor that can be integrated onto robotic platforms for remote monitoring in aquatic field environments [32] and have demonstrated that multilayer EG on SiC(0001) is a stable working electrode for the trace analysis of chemicals in seawater [33]. In this study, we compare multilayer EG, nominally monolayer EG, and hydrogen-intercalated QFS bilayer EG, each with or without an oxygen plasma modification, and evaluate their performance as working electrodes for measuring heavy metal ions in seawater using anodic stripping cyclic square wave voltammetry. It was hypothesized that adding oxygen functional chemical groups to the surface of graphene via oxygen plasma exposure would increase sensitivity by increasing the binding of the heavy metals with the addition of oxygen chelating groups.

Dose–response curves and machine learning algorithms for identification were employed to evaluate the performance of the different EG electrodes. Machine learning models are widely utilized for sample classification of sensor data, and we previously showed their ability to successfully classify electrochemical signatures with high accuracy [33,34]. Here we used dose–response curves and machine learning algorithms to both demonstrate sample automatic identification and as an additional mechanism to evaluate the performance of the different combinations of modifications. 

## 2. Materials and Methods

### 2.1. Materials

Cadmium chloride (CdCl_2_), copper sulfate pentahydrate (CuSO_4_), mercury chloride (HgCl_2_), and lead chloride (PbCl_2_) were purchased from Sigma-Aldrich (St. Louis, MO, USA). Heavy metal salt stock solutions were prepared at 10 mg/mL in distilled water and then diluted to 0.1 mg/mL for testing. For PbCl_2_, a small amount of 1M NaOH and mild heating was used to aid dissolution. Seawater samples were collected at 20 feet below the surface at a latitude of 38.4735 and longitude of −74.89152, off of Ocean City, MD. The conductivity of the seawater sample was 46 mS/cm and the pH was 7.85. The collected seawater was stored at 4 °C.

### 2.2. Epitaxial Graphene Synthesis, Oxygen Plasma Modification, and Surface Characterization

EG was synthesized by sublimation of Si from 8 × 8 mm^2^ SiC substrates (II-VI Inc., Saxonburg, PA, USA) at high temperatures in 100 mbar Ar ambient in a chemical vapor deposition reactor (Axitron/Epigress VP 508) [28,33,35,36,37]. Substrates were first raised to growth temperature under hydrogen flow to remove polishing damage. Monolayer EG and hydrogen-intercalated QFS bilayer EG were grown on semi-insulating, on-axis 6H-SiC(0001) for 20 min at 1595 °C. Hydrogen-intercalated QFS bilayer samples were subsequently annealed at 1050 °C in 900 mbar hydrogen for 1 h [36,37]. Multilayer EG was grown on N^+^, 4H-SiC(0001) 4° offcut towards the [110] direction at 1555 °C for 20 min.

Oxygen plasma modification was performed in a Plasma Preen-II-973 microwave plasma system. The process chamber was evacuated to 20–30 mTorr, then filled to 100 mTorr under ~1.5 scfh oxygen flow. Samples were exposed to 100 W plasma for 15 s.

The graphene samples were characterized by X-ray photoelectron spectroscopy (XPS), atomic force microscopy (AFM), Raman spectroscopy, and van der Pauw Hall measurements before and after oxygen plasma exposure. XPS measurements were made using a Thermo Scientific Nexsa spectrometer with a monochromatic Al-Kα source (1486.68 eV), 400 µm spot size, and flood gun for charge compensation. Survey spectra were acquired using 200 eV pass energy and 1 eV step size. High resolution scans were collected using 20 eV pass energy and 0.1 eV step size. Peak fits were performed in Avantage software using Shirley backgrounds and convolutions of Gaussian-Lorentzian line shapes. The graphene peak of the C 1s scan was fitted using an asymmetric peak shape [38]. Spectra for monolayer and multilayer EG were shifted to the position of the C 1s peak for Si-C bonding at 283.7 eV [28,39,40]. For intercalated EG, a shift to lower binding energy is known to occur for the C 1s Si-C and Si 2p peaks due to a difference in surface band bending after intercalation [39,41,42,43]. Therefore, the spectra for hydrogen-intercalated QFS bilayer EG were shifted to the position of the C 1s SiC peak at 282.6 eV [39,44].

For AFM, a Bruker Dimension FastScan instrument in tapping mode was used. AFM images were analyzed using Gwyddion software. A Thermo DXRxi Raman Microscope was used to obtain 20 × 20 μm^2^ maps of Raman spectra in 0.3 µm x and y stage increments using a 532 nm laser at 9.6 mW and 100× microscope objective. The Raman spectrum of the SiC substrate was acquired and subtracted from the EG spectrum for each sample. Raman peaks were fitted to Lorentzian functions using a linear background in the region of the peak. Scanning electron microscope (SEM) images of hydrogen-intercalated QFS graphene were acquired using a LEO Supra SEM with in-lens secondary electron detector and 5 kV accelerating voltage. Van der Pauw Hall measurements were performed on 8 × 8 mm^2^ monolayer and QFS bilayer EG samples using a home-built system with 2060 Gauss magnet. Carbon on the back surface of the substrate was exfoliated with tape prior to Hall measurements.

### 2.3. Electrochemistry

A custom-built portable potentiostat (the CStat v3.79) was used for all assays reported in this study. This potentiostat has been demonstrated previously for electrochemical detection of multiple compounds, including nitrogen-containing explosives, heavy metal ions, herbicides, pesticides, and industrial chemicals [32,33,34]. Ni/Au (20 nm/50 nm) contact pads were electron-beam evaporated (FerroTec Temescal, Tokyo, Japan) through a foil shadow mask onto all four corners of the 8 × 8 mm^2^ EG sample to facilitate electrical connection to a copper clip wired to the potentiostat. The 8 × 8 mm^2^ EG working electrodes were mounted in a custom-made Teflon cell [45]. The funnel-shaped cell allowed the counter and reference electrodes to be suspended above the graphene. A compact spiral platinum counter electrode (99.9% Pt, Metrohm, Herisau, Switzerland) and an Ag/AgCl reference electrode (Metrohm) were used.

The heavy metal ion determination was made in 1 mL seawater samples, which were placed into the Teflon sample cell. The reference and counter electrodes were inserted into the liquid from above. The solution was mixed to remove any air bubbles. A background cyclic square wave voltammogram was taken as the negative control. The heavy metal ion was added from stock solutions from 1 to 30 μL to give a final concentration range of 100–3000 ppb in metal salt. Concentrations were measured three times and peak heights in the anodic square wave voltammetry were used to generate dose–response curves. Parameters used for cyclic square wave voltammetry included an accumulation step for two minutes at 1 V vs. Ag/AgCl followed by a voltage sweep at a square wave frequency of 17.5 Hz from 1 to −1 V vs. Ag/AgCl, and then a second accumulation step at −1 V vs. Ag/AgCl for two minutes followed by a second voltage sweep at a square wave frequency of 17.5 Hz from −1 to 1 V vs. Ag/AgCl. All electrochemical measurements were made at room temperature.

Dose–response curves were fitted using the nonlinear least squares function in the R stats package [46]. Data was obtained by extracting the maximum anodic stripping peaks and then normalizing by subtracting background using a point selected from 0.512 V vs Ag/AgCl (or data point #880 within the vector created by the concatenated scan). To plot 0 ppb concentration, points from the base of the peak were identified for each heavy metal salt and types and modifications of graphene by a local minimal finding function. Linear, sigmoidal, or hyperbolic equations were used according to which had the lowest mean standard error.

### 2.4. Machine Learning

Training and evaluation of machine learning models for classifying metals was performed as previously described, with some modifications [33,34]. The different models, using Long Short-Term Memory (LSTM), Fully Convolutional Networks (FCN), and variants, were implemented in Keras. Models were trained for 1000 epochs. Each library from the different materials were split into train and test sets at a ratio of 60:40. No data preprocessing was performed prior to training aside from concatenation of cathodic and anodic scans (cathodic followed by anodic). Sample classes were defined by either metal ion or metal salt concentration. Receiver Operation Characteristic (ROC) curves, confusion matrices, and bar plots for visualizing the prediction results were generated using ggplot2 in R [46]. Micro-average area under the curve (AUC) was used as a metric for comparing model and material performance. For final evaluation of the LSTM model on a set of holdout samples, six samples were randomly selected from the total oxygen modified hydrogen-intercalated graphene dataset: Cu_2500, Hg_2500, Hg_3000, Pb_200, Pb_2500, and seawater_0, where the prefix corresponds to the metal ion (or seawater) and the number is the concentration of the metal salt in ppb.

## 3. Results and Discussion

### 3.1. Epitaxial Graphene Characterization and Oxygen Plasma Modification

**Figure 1:**
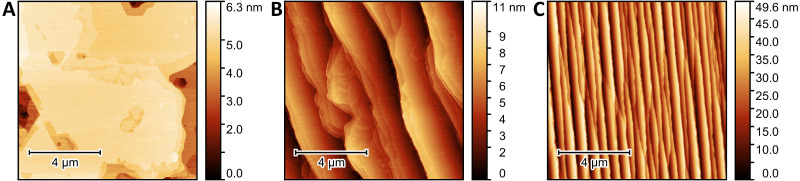
10 × 10 µm^2^ AFM topography images showing morphologies of as-grown (**A**) nominally monolayer EG on on-axis, (**B**) hydrogen-intercalated QFS bilayer EG on on-axis, and (**C**) multilayer EG on 4° off-axis SiC(0001).

AFM images of the as-grown graphene surfaces are shown in Figure 1. Hydrogen-intercalated QFS bilayer EG and multilayer EG (Figure 1B,C, respectively) have the expected stepped morphology of EG synthesized from the Si face of SiC in Ar ambient [35]. Average step heights of hydrogen-intercalated and multilayer samples are 5 nm and 27 nm, respectively, and average terrace widths are 1.5 um and 330 nm, respectively, indicating step bunching has occurred. The higher step density for multilayer EG is a result of the higher offcut angle of its SiC substrate. Monolayer EG samples have a less regular morphology (Figure 1A), which is attributed to the suspected lower offcut angle of the SiC substrate used. For monolayer EG, terrace widths are on the order of 5–10 μm and average step heights are 2–3 nm. The root mean square roughness of the monolayer, hydrogen-intercalated QFS bilayer, and multilayer EG surfaces are 0.7 nm, 1.6 nm, and 9.7 nm, respectively.

**Figure 2:**
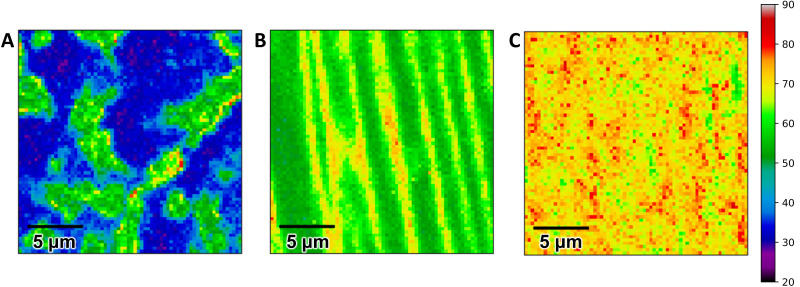
20 × 20 μm^2^ maps of the Raman 2D peak FWHM for unmodified (**A**) monolayer, (**B**) hydrogen-intercalated QFS bilayer, and (**C**) multilayer EG.

The full width at half maximum (FWHM) of the Raman 2D peak is known to increase with EG thickness [47] and can be correlated with the number of graphene layers in the as-grown samples. Figure 2 shows representative Raman maps of the 2D FWHM for each type of unmodified EG and Figure S1 shows histograms of the 2D FWHM distribution with Gaussian fits. Monolayer EG samples consist of single layer graphene on terraces with bilayer or three or more layers at step edges (Figure 2A). The percentage of single layer, bilayer, and three or more layers as determined from Raman mapping ranged from 61–65%, 25–36%, and 0–14%, respectively, based on analysis of 20 × 20 μm^2^ areas taken from four different monolayer EG samples. 

Growth of EG on SiC(0001) is known to be preceded by the formation of a non-conductive interfacial (or buffer) layer consisting of a 6√3 × 6√3 R30˚ surface reconstruction in which ~1/3 of C atoms, arranged in a honeycomb structure, are covalently bonded to Si atoms of the SiC surface [19,48,49]. The hydrogen intercalation process severs the Si and C bonds at the SiC/buffer layer interface, leaving the Si bonds at the SiC surface hydrogen-terminated and converting the buffer layer to an additional graphene layer [19]. Because the buffer layer is only present on the (0001) terraces and not at the step edges, hydrogen-intercalation can result in improved thickness uniformity [37]. Figure 2B shows that the hydrogen-intercalated QFS bilayer EG terraces are comprised of bilayer graphene, and three or more layers are present at the step edges, with percentages ranging from 59–61% bilayer and 38–41% three or more layers. This is in agreement with thickness percent areas calculated by analysis of SEM images of the hydrogen-intercalated samples, which reveal 62% bilayer, 21% three layer, and 17% four layer graphene (Figure S2A,B) [50].

The majority of 2D peak FWHM values of multilayer EG samples are distributed as a Gaussian centered at 70–71 cm^−1^, indicating that the sample is predominately three or more graphene layers (Figure 2C and Figure S1C). While it is difficult to distinguish between three layers and more than three layers using the Raman 2D peak FWHM mapping technique, cross sectional TEM shows that the multilayer EG has four graphene layers on terraces with thicker graphene at step edges (Figure S3).

**Figure 3:**
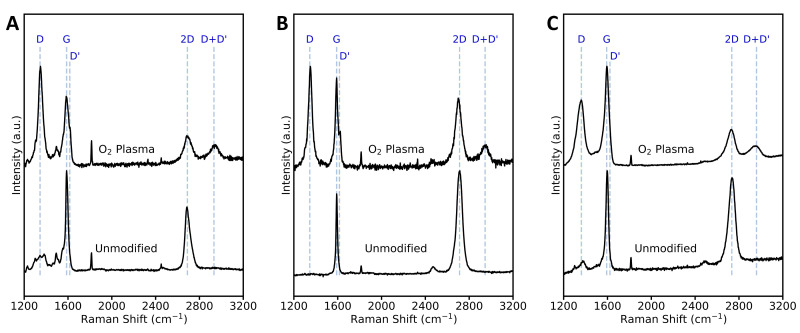
Average Raman spectra over 20 x 20 μm^2^ mapped areas before and after exposure to oxygen plasma for (**A**) monolayer, (**B**) hydrogen-intercalated QFS bilayer, and (**C**) multilayer EG. The spectra of the SiC substrate is subtracted.

Raman spectra of all pixels in the 20 × 20 μm^2^ maps were averaged and are plotted in Figure 3 for the different EG samples before and after oxygen plasma treatment. Prior to oxygen plasma exposure, Raman spectra of all samples show the G and 2D peaks characteristic of graphene located around 1590–1600 cm^−1^ and 2690–2740 cm^−1^, respectively. The average position of the 2D peak increases from monolayer, to QFS bilayer, to multilayer EG. In addition, the spectra for monolayer (Figure 3A) and multilayer EG (Figure 3C) show low intensity, broad peaks between 1200 and 1665 cm^−1^ that are characteristic of the 6√3 buffer layer [51]. The 6√3 buffer layer peaks are not present for QFS bilayer EG (Figure 3B), as the hydrogen intercalation process converted the buffer layer to an additional graphene layer. 

After oxygen plama exposure, Raman spectra for all samples show the presence of D (~1350 cm^−1^), D’ (~1620 cm^−1^), and D + D’ (~2935–2965 cm^−1^) peaks, which require a defect for activation and can arise from defects such as vacancies and sp^3^ hybridization [52,53,54]. The intensity of the D peak can be used to quantify the amount of defects, where the D to G peak intenisty ratio (I_D_:I_G_) initially increases with number of defects for low defect densities then decreases for high defect densities as structural disorder becomes dominant [52,55,56,57]. Additionally, the 2D to G peak intenisty ratio (I_2D_:I_G_) decreases with increasing disorder, while the FWHM of the 2D, G, and D peaks are expected to increase [54,55,58]. For monolayer and hydrogen-intercalated QFS bilayer EG, the I_D_:I_G_ and peak ratio was found to depend upon location on the surface. Figure S4 shows Raman spectra of an oxygen plasma treated, hydrogen-intercalated sample in regions of a terrace and step edge. The terrace has a higher intensity ratio of D to G peaks (I_D_:I_G_ = 1.6) than the step edge (I_D_:I_G_ = 1.0), and the 2D to G peak intensity ratio is higher at the terrace (I_2D_:I_G_ = 0.9) compared to the step (I_2D_:I_G_ = 0.7). The number of graphene layers is greater at the step edges than on the terraces, which may contribute to these observed differences [59,60].

**Figure 4:**
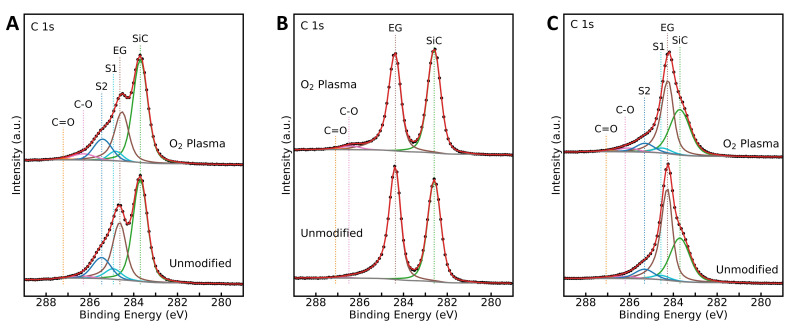
XPS for C 1s comparing oxygen plasma and unmodified samples for (**A**) monolayer EG, (**B**) hydrogen-intercalated QFS bilayer EG, and (**C**) multilayer EG.

XPS data was acquired for the different samples before and after oxygen plasma exposure. Survey scans reveal only oxygen-, silicon-, and carbon-related peaks present for all samples and an increase in O 1s peak intensity after oxygen plasma treatment (Figure S5a–c). The C 1s spectra for monolayer EG (Figure 4A) show peaks assigned to the Si-C bonds in the substrate (“SiC”), sp^2^ carbon in graphene (“EG”), the buffer layer (“S1” and “S2”), C-O bonds, and C=O bonds at ~ 283.7 eV, 284.6 eV, 284.9 eV, and 285.5 eV, 286.3 eV, and 287.2 eV, respectively [28,49,61]. For multilayer EG, the C 1s peak corresponding to sp^2^ graphene is shifted to a lower binding energy (~284.3 eV) (Figure 4C). This shift is consistent with a previous report for multilayer EG [49] and is attributed to the more neutral charge of the topmost graphene layers that are located away from the substrate-graphene interface. For QFS bilayer EG, the buffer layer-related C 1s peaks “S1” and “S2” are not present, indicating successful decoupling of the buffer layer from the substrate (Figure 4B). Additionally, there is increased separation between the C 1 s “SiC” and “EG” peaks, located at 282.6 eV and 284.4 eV, respectively, which is expected of hydrogen-intercalated EG. The shift to lower binding energy of the C 1s “EG” peak is a result of p-type doping after hydrogen intercalation, while the shift to lower binding energy of the C 1s “SiC” and Si 2p peaks is due to surface band bending after intercalation [39,41,42,43].

After oxygen plasma exposure, the percent area of the C 1s peak corresponding to C-O and C=O bonds increased, while that of the “EG” peak decreased for all EG types, suggesting the removal of sp^2^ graphene and formation of C=O and C-O functional groups. Additionally, all samples exposed to oxygen plasma show an increase in O 1s peak intensity (Figure S5d–f). The O 1s spectra were fitted to two peaks assigned to C-O (~533 eV) (epoxy, hydroxyl groups) and C=O (531.8 eV ± 0.13 eV) (carbonyl groups) [28,62,63]. Percent oxygen in the graphene layers was found to increase after oxygen plasma exposure from 1% to 5%, 2% to 6%, and 0.3% to 2%, for monolayer, QFS bilayer, and multilayer EG, respectively. The relatively low percent oxygen for multilayer EG may be due to the greater number of graphene layers for multilayer EG and only the surface layer being modified [64].

Hall measurements were performed on monolayer and hydrogen-intercalated QFS bilayer EG samples using a van der Pauw configuration. Hall measurements were not taken on the multilayer EG samples due to the conductivity of their N^+^ SiC substrate. Prior to oxygen plasma exposure, monolayer EG had a mobility of ~450 cm^2^/V-s and sheet electron concentration of n_sh_ ~ −8 × 10^12^ cm^−2^. Dangling Si bonds between the buffer layer and SiC are known to induce n-type doping in overlying EG and contribute to phonon scattering which lowers the graphene electron mobility. After hydrogen intercalation and removal of the buffer layer, QFS EG exhibits intrinsic p-type conductivity due to induced charge from the bulk polarization field of the SiC substrate [65]. Additionally, due to the lack of phonon scattering, QFS EG has a higher carrier mobility. Prior to oxygen plasma exposure, the QFS bilayer EG samples had an average mobility of 2050 ± 220 cm^2^/V-s and an average sheet hole concentration of p_sh_ =1.2 (±0.3) × 10^13^ cm^−2^. The mobility of the QFS bilayer EG decreased to ~70 cm^2^/V-s after oxygen plasma, while that of monolayer EG was too low to be measured by the Hall measurement system. After oxygen plasma exposure, the sheet resistance of the EG samples increased from ~1800 to 2600 Ω/□ and 260 to 3800 Ω/□ for monolayer and QFS bilayer EG, respectively.

### 3.2. Heavy Metal Electrochemical Detection at Modified Epitaxial Graphene

For the electroanalytical analysis of the spiked seawater samples, we used an inexpensive in-house built portable potentiostat with different types and modifications of EG as a working electrode. The instrumental parameters included a combination of cyclic square wave voltammetry and stripping voltammetry which, for the purpose of developing an automated measurement, works well, since the technique involves an in-situ cleaning of the electrode immediately before use, adsorption of the analyte, and stripping voltammetry for detection. This combination also works well to generate large libraries of electrochemical signatures of environmental contaminates for using machine learning for chemical identification [33,34].

**Figure 5:**
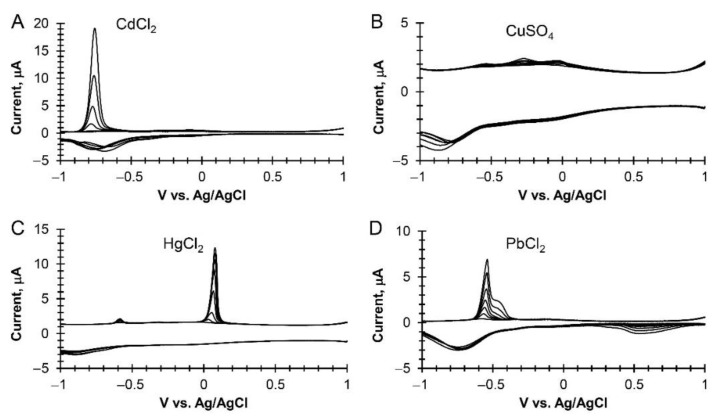
Examples of cyclic square wave voltammograms and the dose–response of heavy metal salts in seawater between 0 to 3 ppm. (**A**) CdCl_2_ at oxygen modified multilayer EG. (**B**) CuSO_4_ at unmodified multilayer EG. (**C**) HgCl_2_ at unmodified hydrogen-intercalated QFS bilayer EG. (**D**) PdCl_2_ at oxygen modified hydrogen-intercalated QFS bilayer EG.

The preliminary work on heavy metal salt detection of CdCl_2_ and PbCl_2_ using unmodified and oxygen plasma-modified monolayer EG performed poorly when compared to other EG types and modifications (Figure S6). The electrode was unstable with repeated scans and gave smaller signals. As a result, the majority of the work was focused on multilayer EG (unmodified and oxygen plasma-modified) and hydrogen-intercalated QFS bilayer EG (unmodified and oxygen-plasma modified) using seawater samples with additions of four heavy metal salts. As shown in Figure 5, for the various heavy metal cations, the cathodic peaks are concomitant with oxygen reduction, and the anodic stripping peaks have their respective peak potentials [33].

**Figure 6:**
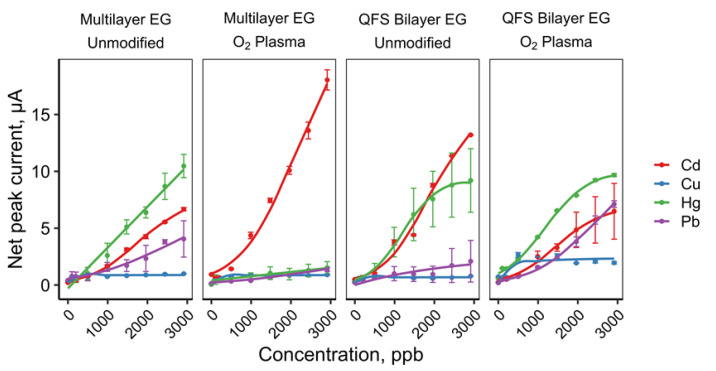
Dose–response curves (ppb in metal salt) for each type of EG and modification.

Using these distinctive peaks for each metal ion from the cyclic square wave voltammograms, the dose–response in current between 100–3000 ppb (in metal salt) for each analyte was plotted in Figure 6 with the fitting parameters listed in Table 1. The dose–response displayed was dependent on the analyte and type of EG used. For both CuSO_4_ and CdCl_2_, shape of the dose–response was consistent for each EG material used, where the best non-linear fits were hyperbolic and sigmoidal curves, respectively. The shape of the dose–responses for HgCl_2_ and PbCl_2_ were more complex, as both metals displayed different responses for either multilayer or hydrogen-intercalated QFS bilayer EG. HgCl_2_ showed a linear response on multilayer EG, but sigmoidal on hydrogen-intercalated QFS bilayer EG. Similarly, PbCl_2_ was observed to have a sigmoidal response on multilayer EG while appearing hyperbolic on hydrogen-intercalated QFS bilayer EG (Figure 6). Whether the EG was oxygen-modified did not appear to influence the response to a great degree in terms of the shape (linear, hyperbolic, or sigmoidal), with the exception of PbCl_2_ at the hydrogen-intercalated oxygen-modified QFS bilayer EG. However, in terms of amplitude of the signal, the multilayer EG with oxygen-modification seemed more selective for CdCl_2_.

A linear response suggests that the dose–curve is strictly dominated by the underlying electrochemistry proportional to the concentration of the heavy metal ion, whereas a hyperbolic response suggests the involvement of a binding isotherm at the surface of the electrode for the heavy metal ion. The sigmoidal response proposes a more complicated mechanism that requires a minimum heavy metal ion concentration to initiate the reduction of the metal at the surface. Both the hyperbolic and sigmoidal responses begin to saturate at high concentration consistent with a limited number of active binding sites at the working electrode. At this point we do not provide a detailed model describing the dose–response with the differences between the EG electrodes (e.g., difference in mobility, surface morphology, number of graphene layers, presence of the buffer layer) or changes after oxygen plasma exposure (e.g., decrease in mobility, increase in sheet resistance, increase in defect concentration, increase in C-O and C=O groups) since no conclusive trends were identified. In the current literature, detailed electrochemical experiments, Raman spectroscopy studies, and DFT calculations point to the importance of specific interactions of the heavy metal with graphene active sites during the reduction of the metal ion [20,21,22,23].

Typical limits of detection for heavy metal ion detection at carbon-based materials under buffered conditions are reported to be in low to sub ppb range and are dependent on the type of carbon and its modification [14,16]. In this study, unbuffered measurements were made directly in seawater which resulted in less sensitivity with measurable signals starting at 100 to 200 ppb, consistent with our previous results [33].

### 3.3. Heavy Metal Identifiction at Modified Epitaxial Graphene with Machine Learning

**Figure 7:**
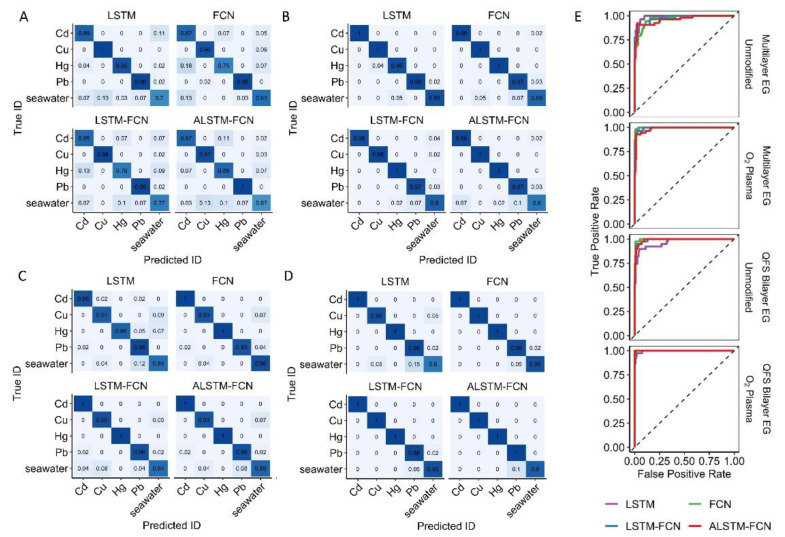
Confusion matrixes comparing the different machine learning models for the library of cyclic square wave voltammograms of seawater spiked with heavy metal salts at different types and modifications of EG. (**A**) Multilayer EG. (**B**) Oxygen-modified multilayer EG. (**C**) Hydrogen-intercalated QFS bilayer EG, and (**D**) Oxygen-modified hydrogen-intercalated QFS bilayer EG. (**E**) Receiver Operating Characteristic (ROC) curves for same data comparing the different machine learning models with the different types and modifications of EG.

The previous strategy for data processing was used prior to training for the machine learning analysis, which included the concatenation of cathodic and anodic scans for each sample, but otherwise the experimental data was not modified [34]. The confusion matrix for the complete set of data at all concentrations, as well as the ROC curves and area under the ROC curves, are shown in Figure 7. Four ML models (LSTM, FCN, LSTM-FCN, and ALSTM-FCN) were each trained on the four different EG types. Each of the metal ions had a high degree of correct identification, with the most consistent misclassification being seawater predicted as metal or vice versa. The results differ widely according to model and EG type. The difference in model performance as the result of material is most clearly seen in the significantly higher correct IDs in oxygen modified hydrogen-intercalated QFS bilayer EG (Figure 7D) relative to unmodified multilayer EG (Figure 7A). The differences in performance between the four models was less clear. Using the area under the curve values of the ROC curves (Figure 7E), the average AUC for the four models for unmodified multilayer EG was 0.972 while the average for oxygen modified hydrogen-intercalated QFS bilayer EG was significantly higher at 0.998. Unmodified hydrogen-intercalated QFS bilayer EG had an average area under the ROC curve of 0.986, and the oxygen modified multilayer EG was 0.995.

**Figure 8:**
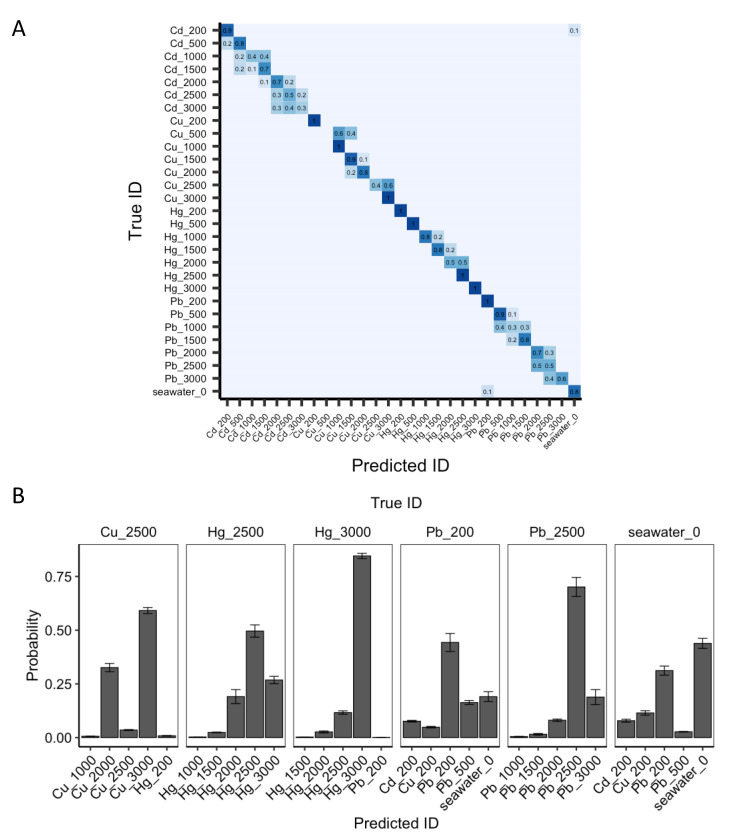
ML model prediction of concentration using oxygen modified hydrogen-intercalated QFS bilayer EG data. (**A**) Confusion matrix results using ALSTM-FCN to predict both metal salt and concentration. The prefix corresponds to the metal (or seawater) and the suffix is the concentration of metal salt in ppb. Probabilities of 0 are removed for clarity. (**B**) Evaluation of holdout dataset. Both identity and concentration of six samples selected at random (true IDs listed at the top) were evaluated for prediction performance. The top five predicted IDs according to probability for each sample are shown.

Using the best-performing model-material pair, ALSTM-FCN and oxygen modified hydrogen-intercalated QFS bilayer EG was evaluated for its ability to classify both metal ion and concentration simultaneously (Figure 8A). In an experiment with repeated shuffle and split cross-validation, the observed performance was promising. With few exceptions, including the misclassifications of 200 ppb PbCl_2_ and CdCl_2_ as seawater or vice versa, each of the classifications was either correct or confined to the metal class. In particular, the model had difficulty distinguishing between higher concentrations (1000, 1500, and 2000 ppb) of CdCl_2_, but none were misassigned as a different metal ion.

Next, the best-performing model-material pair was evaluated on a small set (n = 6) of holdout samples as further confirmation of performance, and to further evaluate the range of probabilities assigned to different compounds (Figure 8B). Here, Cu_2500, Hg_2500, Hg_3000, Pb_200, Pb_2500, and seawater_0 were randomly selected for evaluation and the top five predicted IDs according to probability for each were plotted. Consistent with the confusion matrix, the only misclassification (here, the highest probability assigned) was Cu_2500 as Cu_3000. The only additional issues resulted from low concentrations (200 ppb) and seawater, due to the lower signals from the metal ions, and these samples are still likely to be classified correctly.

## 4. Conclusions

In summary, EG electrodes with different properties were grown, processed, characterized, and then evaluated as working electrodes in an electrochemical sensor for the detection and identification of heavy metal salts in seawater. Graphene growth conditions were varied to produce nominally monolayer EG on on-axis 6H-SiC(0001), hydrogen-intercalated QFS freestanding bilayer EG on on-axis 6H-SiC(0001), and multilayer EG on 4° offcut 4H-SiC(0001). AFM revealed differences in surface morphologies dependent upon SiC offcut angle. 

EG was characterized using Raman spectroscopy, XPS, and Hall measurements before and after exposure to short durations of oxygen plasma. After oxygen plasma modification, Raman spectra exhibit D, D’ and D + D’ peaks associated with defects such as vacancies and sp^3^ hybridization. Additionally, XPS shows an increase in intensity of peaks corresponding to C-O and C=O suggesting the formation of oxygen functional groups. 

The different EG working electrodes, with and without oxygen plasma modification, were mounted in an electrochemical cell and exposed to seawater samples spiked with CdCl_2_, CuSO_4_, HgCl_2_, or PbCl_2_ salts. The dose–response was found to depend upon the metal ion and EG electrode used. Machine learning models were used to identify the type of heavy metal based on cyclic square wave voltammograms. In conclusion, the hydrogen-intercalated, oxygen-plasma modified QFS bilayer EG performed best for correctly identifying heavy metals in seawater.

## Figures and Tables

**Table 1:** Fitting parameters of the dose–response for each analyte.

Analyte	Graphene Type and Modification	Response ^1^	MSE ^2^	Fitting Parameters		
				a	b	x_o_
CdCl_2_	multilayer EG	sigmoidal	0.022	7.454	590.19	1749.080
CdCl_2_	multilayer EG + O_2_	sigmoidal	0.433	22.820	676.20	2088.70
CdCl_2_	QFS bilayer EG	sigmoidal	0.222	15.190	559.59	1834.78
CdCl_2_	QFS bilayer EG + O_2_	sigmoidal	1.260	6.851	1461.96	557.92
CuSO_4_	multilayer EG	hyperbolic	0.018	0.024	0.027	-
CuSO_4_	multilayer EG + O_2_	hyperbolic	0.005	0.014	0.015	-
CuSO_4_	QFS bilayer EG	hyperbolic	0.014	0.051	0.075	-
CuSO_4_	QFS bilayer EG + O_2_	hyperbolic	0.233	0.013	0.005	-
HgCl_2_	multilayer EG	linear	0.493	0.004	−0.296	-
HgCl_2_	multilayer EG + O_2_	linear	0.127	0.0003	0.394	-
HgCl_2_	QFS bilayer EG	sigmoidal	2.737	9.141	408.33	1184.821
HgCl_2_	QFS bilayer EG + O_2_	sigmoidal	0.044	9.990	535.98	1164.120
PbCl_2_	multilayer EG	sigmoidal	0.386	6.825	1008.97	2441.725
PbCl_2_	multilayer EG + O_2_	sigmoidal	0.010	2.347	1104.56	2449.485
PbCl_2_	QFS bilayer EG	hyperbolic	0.717	0.001	0.0003	-
PbCl_2_	QFS bilayer EG + O_2_	sigmoidal	0.042	10.040	699.70	2277.44

^1^ Hyperbolic, f = ax/(1 + bx); sigmoidal, f = a/(1+ e^((−(x − x_o_))/b)); linear, f = ax + b. ^2^ Mean Standard Error.

## Supplementary Materials

The following supporting information can be downloaded at: https://www.mdpi.com/article/10.3390/s22145367/s1, Figure S1: Histograms of Raman 2D peak FWHM maps; Figure S2: SEM image of hydrogen-intercalated QFS bilayer EG; Figure S3: TEM cross section of multilayer EG; Figure S4: Raman spectra of hydrogen-intercalated QFS bilayer EG at step and terrace; Figure S5: XPS survey and O 1s spectra of EG before and after oxygen plasma exposure; Figure S6: Cyclic square wave voltammograms of monolayer EG compared to multilayer and QFS bilayer EG.
