# Supplementary material for: Modifications of Epitaxial Graphene on SiC for the Electrochemical Detection and Identification of Heavy Metal Salts in Seawater
Source: Sensors (Basel). 2022 Jul 19;22(14):5367. doi: 10.3390/s22145367 (PMC9315748; doi:10.3390/s22145367)
Supplement: Supplementary file 1 [file sensors-22-05367-s001.zip › sensors-1809803-supplementary.pdf]

Supporting Information

Modifications of Epitaxial Graphene on SiC for the Electrochemical Detection and Identification of Heavy Metal Salts in Seawater

Jenifer R. Hajzus [1], Lisa C. Shriver-Lake [2], Scott N. Dean [2], Jeffrey S. Erickson [2], Daniel Zabetakis [2], Joel Golden [2], Daniel J. Pennachio [3], Rachael L. Myers-Ward [2] and Scott A. Trammell [2,*]

[1] ASEE Research Associate at the U.S. Naval Research Laboratory, Washington, DC 20375, USA; jenifer.hajzus.ctr@nrl.navy.mil (J.R.H.)
[2] U.S. Naval Research Laboratory, 4555 Overlook Avenue SW, Washington, DC 20375, USA; lisa.shriver-lake@nrl.navy.mil (L.C.S.-L.); scott.dean@nrl.navy.mil (S.N.D.); jeffrey.erickson@nrl.navy.mil (J.S.E.); daniel.zabetakis@nrl.navy.mil (D.Z.); joel.golden@nrl.navy.mil (J.G.); rachael.myers-ward@nrl.navy.mil (R.L.M.-W.)
[3] NRC Research Associate at the U.S. Naval Research Laboratory, Washington, DC 20375, USA; daniel.pennachio.ctr@nrl.navy.mil (D.J.P.)
* Correspondence: scott.trammell@nrl.navy.mil; Tel.: +1-202-404-6063

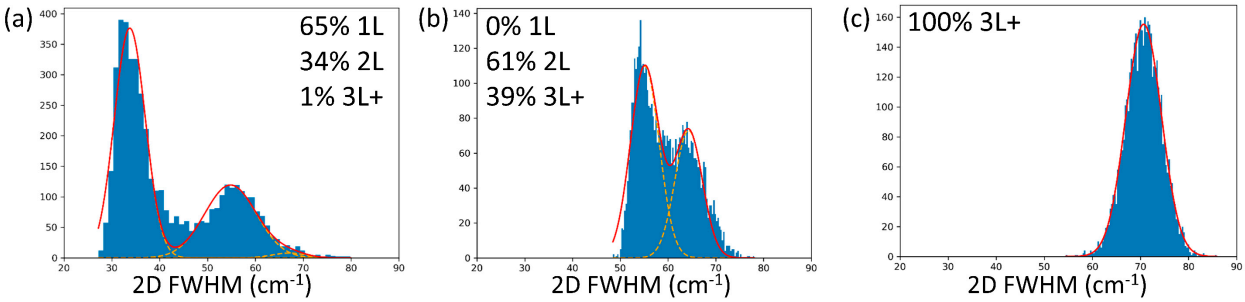

Figure S1. Histograms of the Raman 2D peak FWHM for maps shown in Figure 2 for unmodified (a) monolayer (b) hydrogen-intercalated QFS bilayer, and (c) multilayer epitaxial graphene (EG).

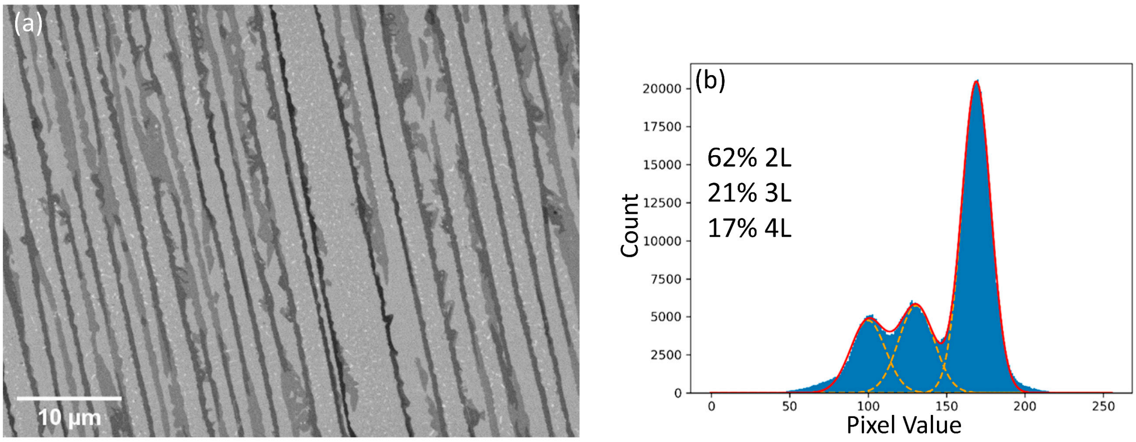

Figure S2. (a) Example SEM image of hydrogen intercalated, quasi-freestanding bilayer EG. (b) Histogram of pixel values of the SEM image shown in Fig S1a and Gaussian fits to the three different peaks, which correspond to regions of two layer (2L), three layer (3L), and four layer (4L) graphene. Area ratios of the Gaussian peaks were used to calculate the percent area of each thickness.

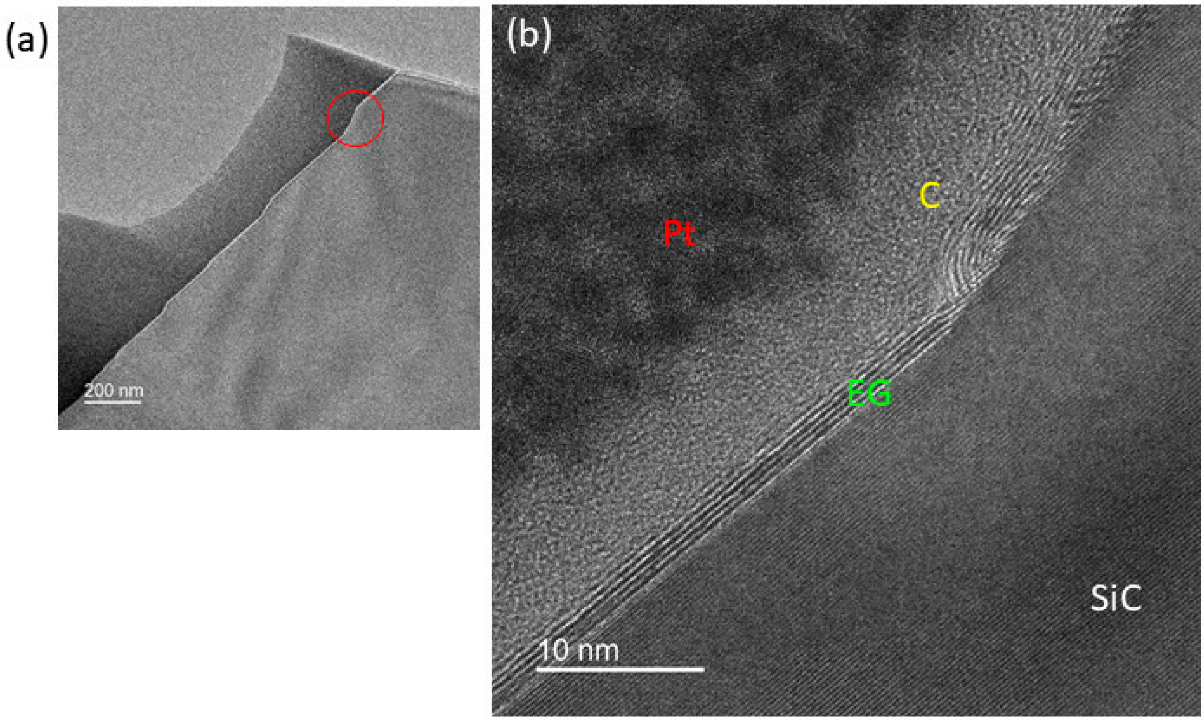

Figure S3. TEM cross section of multilayer EG, showing four layer graphene on the terrace and thicker graphene at the step edge.

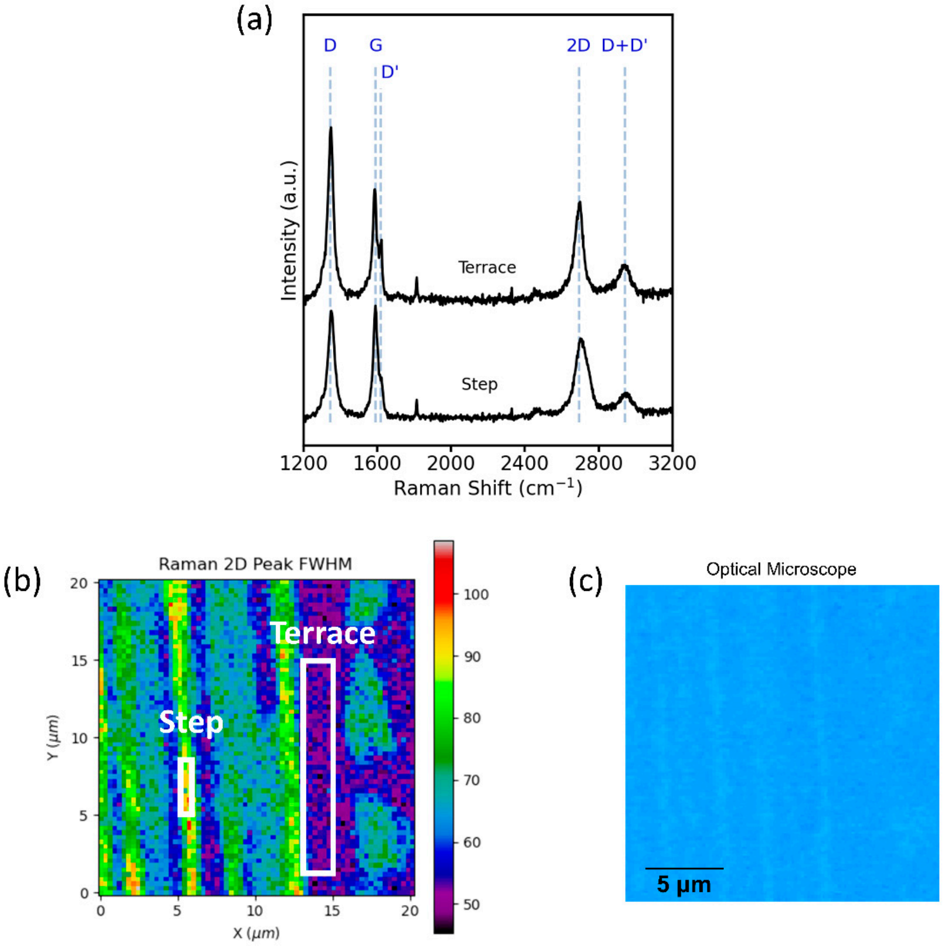

Figure S4. (a) Raman spectra of oxygen plasma-treated, H-intercalated, QFS bilayer EG for a terrace and step edge showing differences in spectra between the two regions. (b) Map of Raman 2D peak FWHM, showing the areas over which the spectra plotted in (a) were averaged for the terrace region and step regions. (c) Optical microscope image of the mapped region in (b) showing the location of step edges.

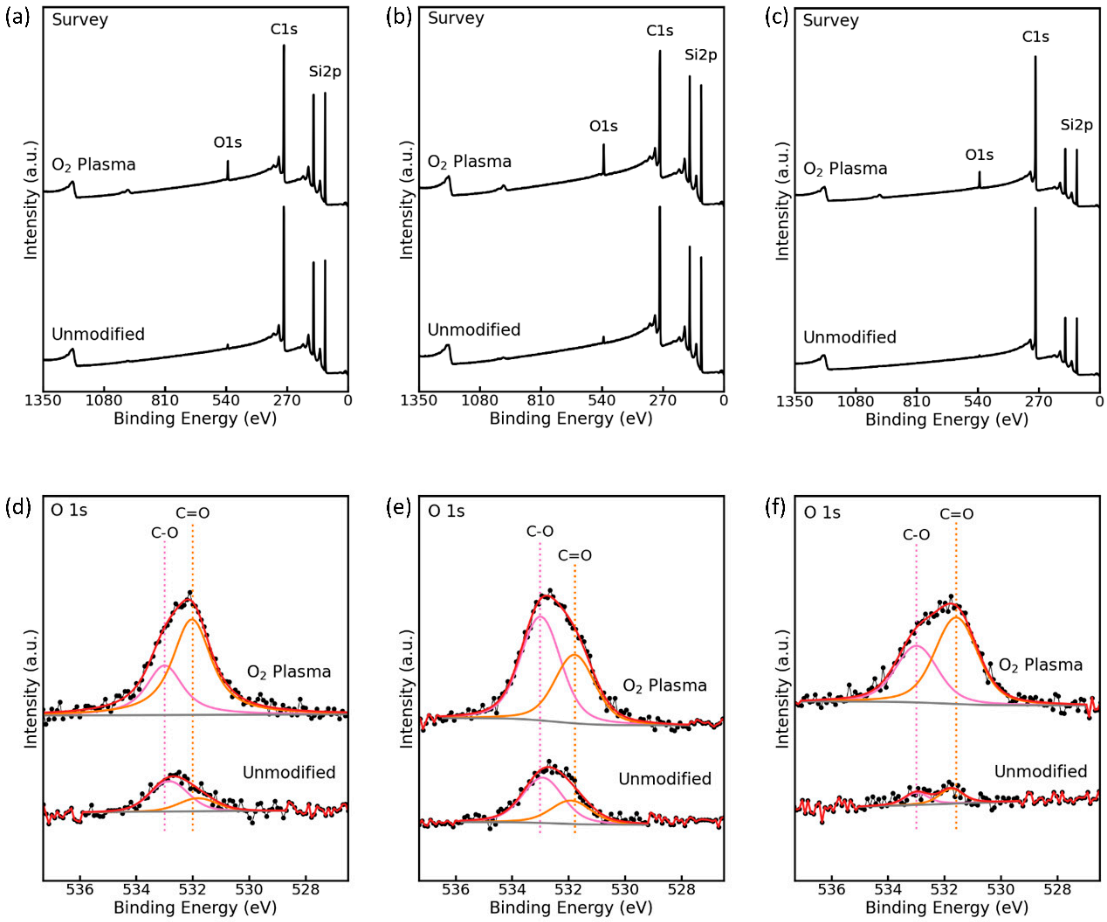

Figure S5. (a-c) XPS survey spectra before and after oxygen plasma exposure for (a) monolayer, (b) H-intercalated QFS bilayer, and (c) multilayer EG. (d-f) High resolution O 1s scan before and after oxygen plasma exposure for (d) monolayer, (e) H-intercalated QFS bilayer, and (f) multilayer EG.

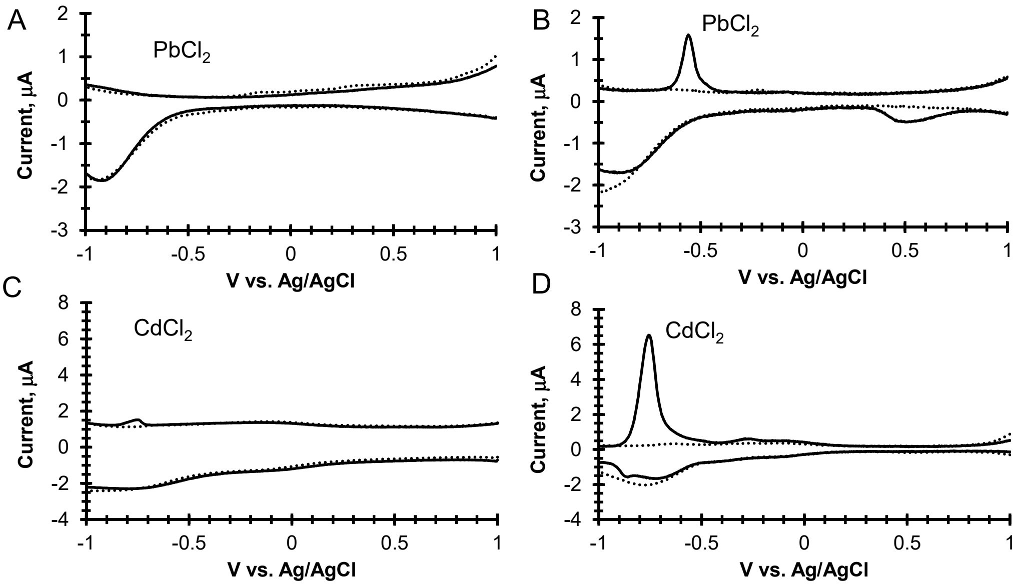

Figure S6. Cyclic square wave voltammograms. Monolayer EG (A) and multi-layer EG (B) in seawater (dotted line) and with the addition of 1.5 ppm $PbCl_2$ (solid line), oxygen modified Monolayer EG (C) and oxygen modified H-intercalated QFS bilayer EG (D) in seawater (dotted line) 1 and with the addition of 2 ppm $CdCl_2$.
